# Ellipsometric Characterization of Network Topology Transition in Vitrimers

**DOI:** 10.1021/acsmacrolett.5c00640

**Published:** 2025-11-14

**Authors:** Yuming Wang, Jaylen Davis, Travis L. Thornell, Sergei Nazarenko, Derek L. Patton, Yoan C. Simon, Zhe Qiang

**Affiliations:** † School of Polymer Science and Engineering, 5104 University of Southern Mississippi, Hattiesburg, Mississippi 39406, United States; ‡ Environmental Laboratory, 57629 U.S. Army Engineer Research and Development Center, Vicksburg, Mississippi 39180, United States; § Geotechnical and Structures Laboratory, U.S. Army Engineer Research and Development Center, Vicksburg, Mississippi 39180, United States; ∥ School of Molecular Sciences, Arizona State University, Physical Sciences Center PSD 104, Tempe, Arizona 85287, United States

## Abstract

Vitrimers are an emerging class of covalent adaptable networks, which can be reprocessed at elevated temperatures while preserving crosslinking density. In these systems, the onset temperature of bond exchange is often dubbed “topology freezing transition temperature (*T*_v_)” and is characterized by a sharp reduction in material viscosity. Here, we provide a universal and external stress-free method to determine *T*_*v*_ in submicrometer (<μm) supported films, by measuring their thickness change as a function of temperature by ellipsometry. This study investigated a range of vitrimer systems, including catalyst-free, externally catalyzed, and internally catalyzed networks, to confirm the general applicability of our approach. We demonstrate the high sensitivity of ellipsometry in detecting changes in the apparent thermal expansion behaviors of vitrimer films, specifically linked to the onset of bond exchange in vitrimers, which is distinguished from most other methods that primarily capture macroscopic thermomechanical behaviors. Our results also suggest that the mechanism by which ellipsometry reveals the *T*_v_ in vitrimers is governed by their change in relaxation dynamics, which are fundamentally distinct from the thermodynamically driven glass transition observed in conventional polymers. We believe the ellipsometric method can not only streamline the characterization of *T*_v_ in vitrimers but also provide deeper insights into their dynamic exchange mechanisms by distinguishing between their microscopic and macroscopic properties.

## Graphical abstract

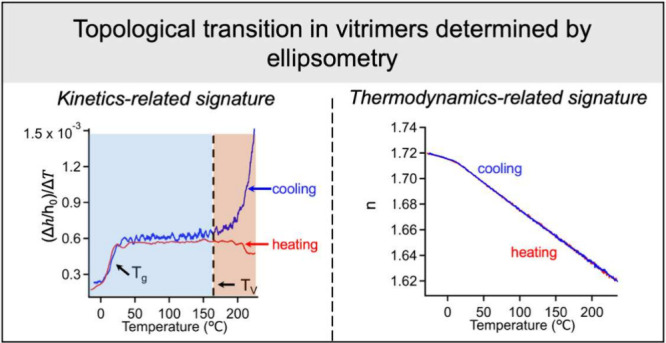

Vitrimers have garnered significant attention in the polymer community over the past decade due to their unique capability to exhibit reduced viscosity at elevated temperatures while maintaining a covalent network structure.^1–6^ In these polymers, the topological rearrangement of the network typically can occur at a characteristic temperature, often referred to as the topology freezing transition temperature (*T*_v_).^7^ Specifically, when the material is below *T*_v_, it behaves similarly to traditional crosslinked materials.^1,8,9^ Upon heating above both *T*_v_ and *T*_g_ (glass transition temperature), the network becomes dynamic and reversible, allowing for flow and reprocessing. This unique ability of vitrimers makes them particularly promising for many advanced applications such as self-healing materials^10–12^ and reprocessable thermosets,^13–15^ providing potential to tackle challenges in material circularity. Notably, characterization of *T*_v_ is crucial for the rational implementation of vitrimer materials. Conventional methods for understanding *T*_v_, such as stress relaxation,^15–19^ creep measurements,^20–22^ and rheology^18,23–26^ typically focus on determining macroscopic material kinetics with the presence of external forces.^27^ While these techniques provide valuable insights into the temperature- and/or strain-dependent thermomechanical responsess of vitrimers, external forces applied during measurements could impact polymer relaxation behaviors and possibly the activation energy required for dynamic bond exchange occurring,^28^ potentially leading to discrepancies in the observed *T*_v_ across different conditions.^21^ Several recent efforts have demonstrated external stress-free measurement approaches to characterize *T*_v_, such as using aggregation-induced emission (AIE) dyes to detect sample free volume,^29^ small-angle X-ray scattering (SAXS) to probe mesoscale structural rearrangements,^30,31^ and temperature-modulated optical refractometry to quantify changes in refractive index as materials surpass *T*_v_.^32^ However, these methods seem to lead to differing conclusions regarding the mechanisms underlying *T*_v_ and their influence on the thermodynamic and kinetic properties of vitrimers, leaving important questions open for the field to address.

Furthermore, most methods for determining *T*_v_ have to date been limited to bulk material samples, making them less suitable for studying vitrimer thin films, which are becoming increasingly important in applications such as flexible electronics,^33–35^ coatings,^36–38^ and biomedical devices.^39–41^ There is therefore a strong need to develop alternative methods to directly access the change in network topology rearrangement of vitrimer films. Ellipsometry is a well-established method to study thermal transitions in polymer thin films.^42–46^ For instance, using this method, the *T*_g_ of polymer films can be determined by quantifying the change in thickness or thermal expansion coefficients between the rubbery and glassy states.^47–49^ We propose to use ellipsometry to directly determine the *T*_v_ of vitrimers by characterizing film thickness as a function of temperature. This study focuses on understanding vitrimer systems in which *T*_v_ is higher than *T*_g_. We established that, above *T*_v_, bond exchange of crosslinkers mediates network relaxation and reduces viscosity, which in turn significantly alter the internal surface tension, leading to a distinct thermal expansion response in vitrimer thin films. This behavior is consistent across all vitrimer systems studied and is absent in permanently crosslinked networks. Our method eliminates the need of using external forces or guest molecules for enabling direct characterization of *T*_*v*_ in thin films with minimal material consumption.

This study first focused on an aromatic disulfide vitrimer as a model system, prepared by crosslinking poly­(ethylene glycol) diglycidyl ether (PEGDGE, molecular weight is 200 g/mol) with 4,4′-aminophenyl disulfide (4-AFD) via epoxide–amine ring-opening polymerization (Figure 1(A)). In this network, embedded S–S linkages provide dynamic covalent crosslinks that undergo thermally activated disulfide exchange above *T*_v_ with the absence of a catalyst,^50^ allowing topology rearrangement while preserving crosslink density. Bulk samples exhibit a *T*_g_ of approximately 10 °C, as determined by differential scanning calorimetry (DSC, Figure 1(B)). Reaction completion in thin films was verified by FTIR (disappearance of the aliphatic epoxide ring band near ∼910 cm^–1^ and increase of −OH signal near ∼3350 cm^–1^, Figure S1) and gel-fraction measurements; an insoluble content of ∼70% was achieved after 1 h of curing at 150 °C (Figure S2). Figure 1(C) presents the non-isothermal creep results for bulk samples under an applied stress of 5 kPa, from which the *T*_v_ of bulk samples was determined to be at approximately 145 °C. Note that in some previous studies, *T*_v_ was defined as the temperature at which the strain begins to deviate from the linear trend at the end rubbery plateau range.^1,21^ Here, we chose to determine *T*_v_ as the intersection point of linear fits applied to distinct temperature regimes.^16^

**Figure 1:**
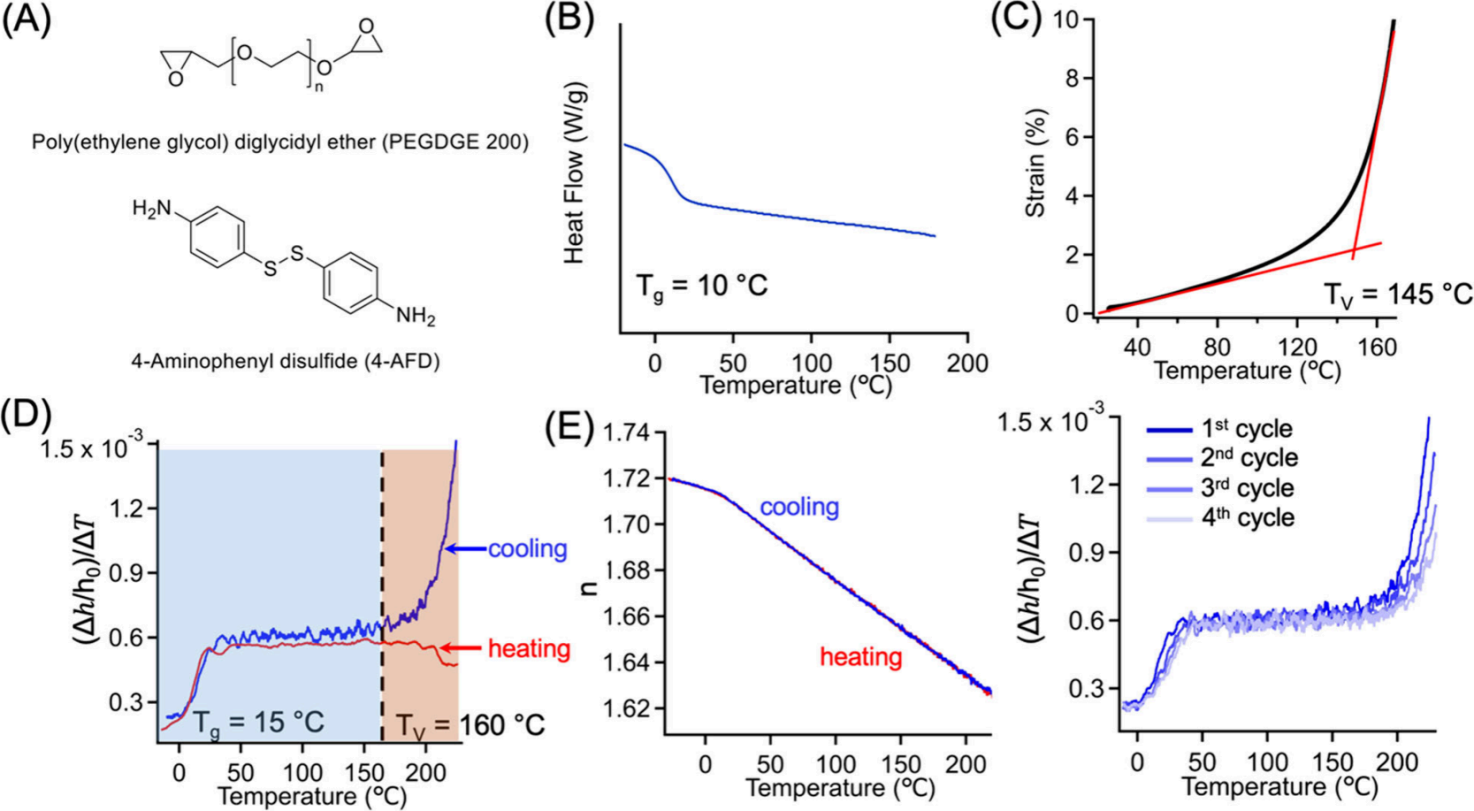
(A) Chemical structure of PEGDGE and 4-aminophenyl disulfide for preparing catalyst-free vitrimers. (B) DSC results (obtained from the 2nd heating cycle) for bulk vitrimer samples. (C) Non-isothermal creep measurement for disulfide-based vitrimer with an applied stress of 5 kPa. (D) Plot of $\frac{\Delta h/h_{0}}{\Delta T}$ vs temperature for disulfide-based vitrimer films obtained during cooling (blue) and heating (red) cycles. The change from light blue region to orange region represents the transition at *T*_v_. (E) Refractive index (*n*) as a function of temperature for disulfide-based vitrimer films derived obtained during cooling (blue) and heating (red) cycle. (F) Plot of $\frac{\Delta h/h_{0}}{\Delta T}$ vs temperature for disulfide-based vitrimer films as a function of cycle number. The vitrimer film thickness was approximately 200 nm.

We aimed to demonstrate that ellipsometry method can be used to characterize *T*_v_ in thin films of vitrimers without the need for external forces, simply by analyzing the changes in $\frac{\Delta h/h_{0}}{\Delta T}$ as a function of temperature using eq 1 below,

1 $$\frac{\Delta h/h_{0}}{\Delta T}=\frac{h\left(T+\frac{\Delta T}{2}\right)-h\left(T-\frac{\Delta T}{2}\right)}{h_{0}\Delta T}$$

where $\frac{\Delta h/h_{0}}{\Delta T}$ is the temperature-dependent normalized apparent thermal expansivity of polymer thin films, *h* is the temperature-dependent thickness, *h*_0_ is the initial polymer film thickness, and Δ*T* is the differentiation range, which was set to be 5 °C. Ellipsometry is a well-established method to detect thermal transitions in polymer thin films, including widely used for *T*_g_ characterization, which can provide reliable data with minimal sample consumption. As shown in Figure 1(D), we observed two distinct changes in the plot of $\frac{\Delta h/h_{0}}{\Delta T}$ versus temperature, corresponding to the *T*_g_ and *T*_v_ at 15 and 160 °C, respectively. The polymer thin film *T*_g_ is comparable with DSC results, indicating that a film thickness of approximately 200 nm does not lead to confinement effects that significantly alter polymer segmental dynamics. Notably, the apparent thermal expansion response is reversible and consistent across heating and cooling near *T*_g_, but it differs markedly in the vicinity of *T*_v_. Upon heating above *T*_v_, $\frac{\Delta h/h_{0}}{\Delta T}$ decreases because thermally activated bond exchange drives internal-surface-tension–mediated contraction/shrinkage (unidirectional and out-of-plane) that opposes thermal expansion, lowering the apparent expansion coefficient. During cooling to below *T*_v_, $\frac{\Delta h/h_{0}}{\Delta T}$ drops more substantially, as intrinsic thermal contraction and the bond exchange-induced vitrimer shrinkage established above *T*_v_ act in concert. This competition on heating and cooperation on cooling leads to the observed difference around *T*_v_, while dimensional shrinkage upon surpassing *T*_v_ is a well-documented phenomenon in vitrimer systems,^27,32^ and our thermomechanical analysis (TMA) results similarly show a sharp reduction in bulk sample dimensions above *T*_v_ (Figure S3).

These results of vitrimer films shown in Figure 1D are intriguing and suggest that the mechanism probed by ellipsometry for characterizing *T*_v_ may be fundamentally distinct from that underlying *T*_g_. It is important to emphasize that efforts to fully understand the topological transition of vitrimers and its impact on their free volume and apparent thermal expansion behavior are still ongoing. Particularly, molecular dynamics simulations with reactive force fields performed by Perego et al.,^52^ found that exceeding *T*_v_ in vitrimers leads to a higher thermal expansion coefficient (α­(T)) compared to permanently cross-linked systems. This increase was attributed to bond exchange processes generating additional free volume and thus a larger specific volume. By contrast, permanently crosslinked systems exhibited only minimal changes in α­(T) with increasing temperature. These simulation findings are consistent with some experimental studies, such as using AIE fluorophores to assess local free volume within samples above *T*_*v*_.^29^ However, other reports potentially challenged this interpretation. For example, scattering methods have been used to probe vitrimer structure across different length scales. It was found that polymer chains exhibited the same apparent thermal expansion coefficient across the entire viscoelastic region, while the network structural transition surpassing *T*_v_ emerges only at the mesoscale.^30^

To further understand the mechanistic origin of *T*_v_ effects on vitrimer films, we determined their refractive index as a function of temperature from ellipsometry (Figure 1(E)). When the temperature is higher than *T*_*g*_, it increased linearly with temperature and showed no anomaly upon crossing *T*_v_, indicating very limited free volume change within our measurement window. This result supports the interpretation that the apparent changes in thermal expansion behavior of vitrimer films arise primarily from relaxation kinetics (i.e., reduced viscosity) and bond exchange-mediated internal surface tension, rather than from thermodynamic free volume variations. It is possible that any transient free volume fluctuations were likely not resolved if bond-exchange kinetics are sufficiently fast to appear quasi-equilibrated at our temporal and spatial resolution. Notably, our observations seem to be similar to a recent report. Specifically, Andreas et al.^32^ employed TMA and temperature-modulated optical refractometry (TMOR) to characterize the α­(*T*) of vitrimers as a function of temperature. Their TMA results from cooling showed a clear increase in apparent thermal expansivity upon surpassing *T*_v_, when materials transited from viscoelastic solid state to viscoelastic liquid state, agreeing well with our data shown in Figure 1(D). In contrast, TMOR measurements indicated a nearly constant α­(*T*) across *T*_*v*_ for bulk samples, suggesting a minimal change in the sample free volume, which is consistent with our findings in Figure 1(E). Here, we were able to obtain both temperature-dependent thermal-expansion behavior and refractive index from the same measurement, supporting that relaxation kinetics—rather than thermodynamic changes—can provide the most sensitive signals for characterizing the *T*_v_. Moreover, Figure 1(F) shows the changes in $\frac{\Delta h/h_{0}}{\Delta T}$ as a function of temperature over successive heating–cooling cycles. These thermal cycles do not alter the *T*_v_ observed in our system; however, when above *T*_v_, the magnitude of temperature-dependent change in $\frac{\Delta h/h_{0}}{\Delta T}$ gradually decreases upon repeated cycling. From the refractive index–temperature plots (Figure S4), we observed complete reversibility of the vitrimer films after four consecutive heating–cooling cycles, as further supported by the unchanged *T*_g_ values shown in Figure 1(F). Therefore, the reduced apparent thermal expansion behavior in disulfide-based vitrimers can be attributed to irreversible sample deformation accumulated during repeated measurements.

We then investigated an established catalytic vitrimer systems using DGEBA and sebacic acid (Figure 2(A) and (B)) with TBD as a catalyst,^20^ of which *T*_v_ has been extensively measured by various research groups using different techniques,^16,20,21,27^ making it a suitable benchmark system. Specifically, TBD can promote transesterification at elevated temperatures via a dual-hydrogen bond activation, in which one nitrogen coordinates to the proton of the alcohol and the proton of the secondary amine coordinates to the oxygen of the carbonyl groups.^53–55^ To determine the optimal curing conditions for the DGEBA-sebacic acid network, we performed DSC on the uncured bulk monomer mixture containing 5 mol % TBD. The exothermic peak in the corresponding thermogram (Figure S5­(A), red) was around 170–180 °C.^20,21,27^ The DSC thermogram of the cured samples show the complete disappearance of the exothermic peak (Figure S5­(A)), indicating a completed reaction. FTIR (Figure S5­(B)) further validated the effectiveness of the selected cure conditions through the appearance of stretching bands at 3500 and 1740 cm^–1^, respectively, corresponding to the newly formed hydroxy groups and the carbonyl of ester groups after cross-linking. Concomitantly, the band around 1700 cm^–1^ corresponding to C=O stretching disappeared. Together, these results suggest successful esterification between carboxylic acid and epoxy groups in our model systems. When applied to thin films, curing for 15 min yielded a plateau in the insoluble content, reaching approximately 85% (based on film thickness, Figure S6). To confirm completed network formation under these conditions, we further examined the *T*_g_ as a function of curing time and found that films cured for 15 min at 180 °C exhibited an identical *T*_g_ to those cured for 2 h. This result indicates that a 15 min curing duration was sufficient and therefore adopted as the optimal condition for preparing DGEBA–sebacic acid vitrimer thin films. DSC thermograms of the bulk vitrimer samples (Figure 2(C)) at all catalyst loadings (1 to 5 mol %) showed a constant *T*_g_ around 31−33 °C, indicating that TBD does not act as a plasticizer to influence polymer segmental mobility.

**Figure 2:**
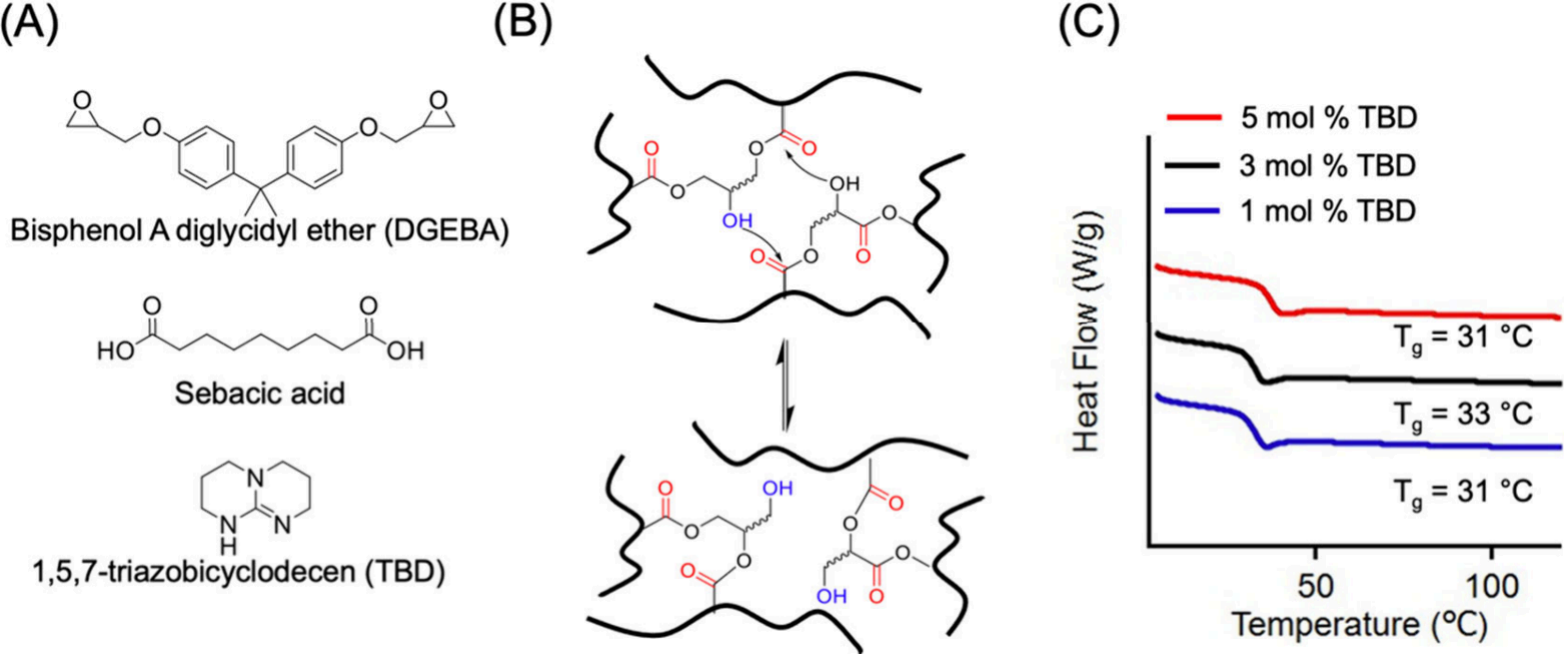
(A) Chemical structure of DGEBA, sebacic acid, and TBD (serving as a catalyst). (B) A simplified illustration of the topological bond exchange via transesterification mechanism. (C) DSC results (obtained from the 2nd heating cycle) for bulk vitrimer samples containing 1, 3, and 5 mol % TBD, respectively.

Figure S8 presents the non-isothermal creep results for bulk samples of the DGEBA-sebacic acid vitrimers under 15 kPa. As the TBD loading increased from 1 mol % to 5 mol %, the *T*_v_ observed from nonisothermal creep in these vitrimer systems decreased from 247 to 207 °C, consistent with literature precedents quantitatively.^16^ We note that the change in *T*_v_ with varying TBD amounts is often attributed to altered vitrimer creep relaxation kinetics and flow behaviors, as captured by thermomechanical measurements.^8^ Increasing catalyst loading can result in faster and/or more frequent bond exchange at the crosslinks, reducing the characteristic relaxation time of the corresponding networks. Furthermore, Li et al. demonstrated that dynamic bonds may be sensitive to the magnitude of the applied force during measurements, with a larger force being able to further reduce the *T*_v_ characterized by material flow behaviors.^15^ In our system, we also observed that changes in external forces impacted the *T*_v_ determined by non-isothermal creep (Figure S9). When the stress was increased from 10 to 50 kPa, the *T*_v_ of the 5 mol % TBD-loaded sample decreased significantly from 220 to 179 °C; our TMA result revealed a *T*_v_ of 205 °C for this sample, suggesting the *T*_v_ value could slightly vary depending on the characterization technique used (Figure S10). Similarly, Kaiser and co-workers examined the impact of external forces on the *T*_v_ of epoxy vitrimers with various acids and catalysts. They observed that *T*_v_,^21^ measured by non-isothermal creep, consistently decreased with increasing applied stress in all vitrimer systems, reaching a plateau at high stress levels.

While previous studies have shown that increasing catalyst loading may reduce the *T*_v_ of vitrimer networks, which was observed in our bulk samples via non-isothermal creep measurements (Figure S8), our ellipsometry data (Figure 3) indicates no noticeable change in the *T*_v_ of vitrimers (between 208 and 211 °C) with increased catalyst loading from 1 to 5 mol %. We believe this can be because conventional thermomechanical-based measurements characterize topological transitions in vitrimers by evaluating material flow activation energies and viscoelastic behaviors, which are often collectively governed by bond exchange dynamics, segmental mobility, crosslinking density, and network heterogeneity. Our ellipsometry measurements could more directly capture intrinsic material responses (e.g., the onset temperature for bond exchange), under which the influence of catalyst loading becomes significantly less pronounced. These results also indicate when above *T*_v_, films with 1 mol % TBD possess a lower apparent thermal expansion coefficient, in contrast to the comparable values observed for 3 mol % and 5 mol % TBD. This trend implies that the bond-exchange-mediated relaxation kinetics in vitrimer thin films are enhanced once the catalyst loading surpasses roughly 3 mol %. We note that Yang and co-workers reported a similar observation to ours: using AIE fluorophore as a guest molecule to probe free volume changes, they found unchanged *T*_v_ values were found despite varying catalyst loading in transesterification-based vitrimer networks.^34^ Interestingly, like here, their approach does not require external forces. Collectively, these findings can explain why *T*_v_ values remained consistent in our measurements, despite different catalyst loading content. Moreover, we prepared a model permanently cross-linked polymer film from 4,4’-methylenedianiline and 1,4-butanediol diglycidyl ether in a 1:2 molar ratio (Figures S11). The film exhibits a *T*_g_ around 50 °C (Figure S12) similar to *T*_g_ characterized by DSC measurements (Figure S11­(B)). No distinct change in $\frac{\Delta h/h_{0}}{\Delta T}$ was observed at temperatures above *T*_g_, and only a minimal increase was detected, which aligns with previously reported simulation results.^52^

**Figure 3:**
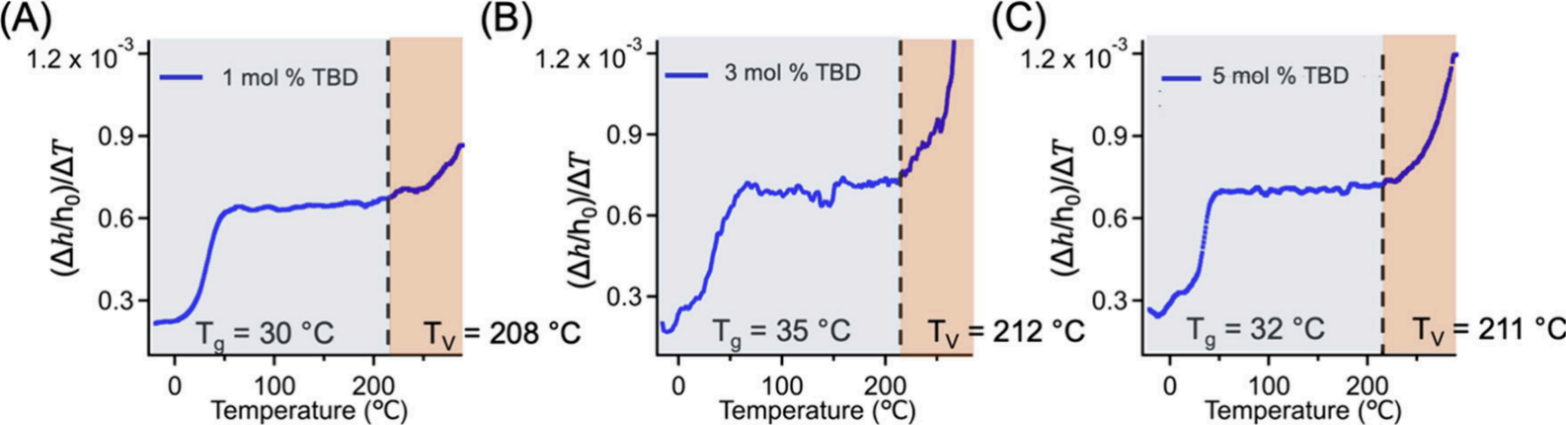
$\frac{\Delta h/h_{0}}{\Delta T}$ vs temperature plot of DGEBA-sebacic acid vitrimer systems with (A) 1 mol %, (B) 3 mol %, and (C) 5 mol % TBD. The change from blue to red represents the transition at *T*_v_. All films have a thickness of approximately 150 nm.

We further studied the effect of cooling rate on the apparent *T*_v_ measured by ellipsometry, using 5 mol % TBD-loaded vitrimer films as a model system. As shown in Figure S13, changing the cooling rate from 10 to 2 °C/min does not have a noticable impact on *T*_v_, slightly increasing from 209 to 211 °C. Moreover, multiple heat–cool cycles (from −30 to 300 °C) were performed on the same sample to assess how thermal history affects both *T*_g_ and *T*_v_. Here, a 5 mol % TBD-loaded film was subjected to a total of 3 heating and cooling cycles at a rate of 5 °C/min. During the third cycle (Figure S14), *T*_g_ remained largely unchanged, while the *T*_v_ transition became less pronounced. Because this effect was more pronounced than in the disulfide vitrimer model system, we attribute it to the thermal degradation and loss of TBD catalytic activity during repeated heating, as previously reported.^56^ Moreover, we investigated the impact of film casting method on *T*_v_ measurements. Specifically, vitrimer films (∼150 nm) were prepared using a dip casting method, employing the same solution used for spin-casting. Ellipsometry curves indicated that both *T*_g_ (around 30 °C) and *T*_v_ (around 210 °C) were similar regardless of whether the film was spin-coated on the substate or dip-coated (Figure S15).

To further demonstrate the versatility of this method, we studied additional benzoxazine-based vitrimers (Figure 4(A–C)), where dynamic bond exchange in the cross-linking units is self-catalyzed (Figure S16). Aminopropyl diethylamine (AP) and monoethanolamine (MEA) were used as precursors for vitrimer samples, while furfurylamine (FFA) was served as the precursor for preparing permanently cross-linked thermoset system. As presented in Figure S17 and Figure 4(D–F), ellipsometry measurements demonstrate a distinct *T*_v_ transition in the $\frac{\Delta h/h_{0}}{\Delta T}$ –temperature plots for both vitrimer systems, with *T*_v_ observed at approximately 124 °C for AP and 121 °C for MEA, respectively. These transitions correspond to the onset of dynamic bond exchange in the systems. In contrast, the FFA thermoset exhibits a linear relationship between thickness and temperature, consistent with its rubbery state behavior and indicates the absence of dynamic bond exchange. Our ellipsometry data corroborates the nonisothermal creep results (Figure S18), with *T*_v_ values of 139 and 150 °C for AP and MEA respectively. This consistency confirms the reliability of ellipsometric measurement method in detecting the topology freezing transition, as the FFA results further confirm that our method effectively differentiates between dynamic vitrimer networks and permanently cross-linked thermosets.

**Figure 4:**
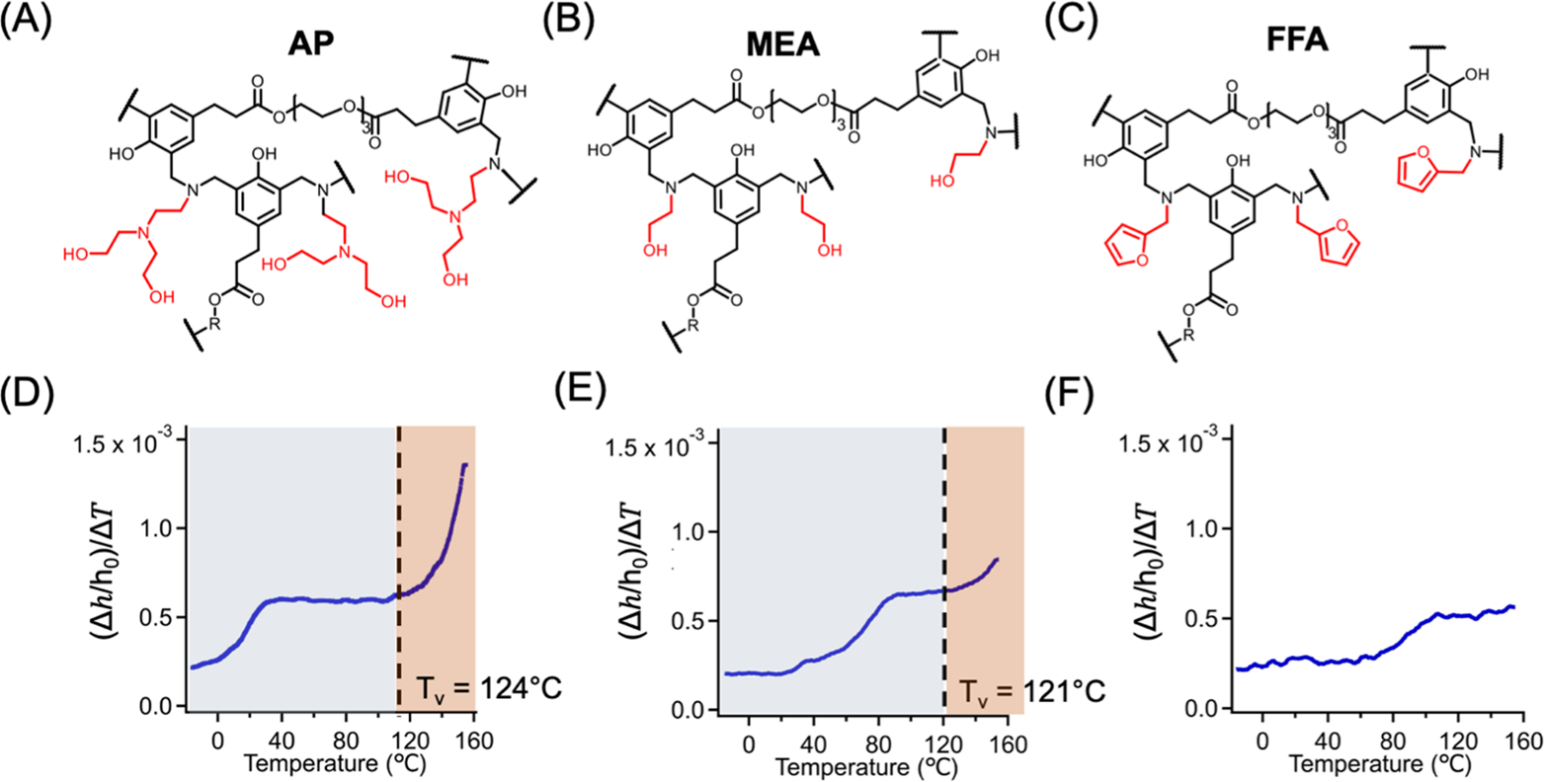
Chemical structure of different cross-linked networks, including vitrimers prepared from (A) aminopropyl diethylamine (AP), (B) monoethanolamine (MEA) precursors, and (C) permanently crosslinked network prepared from furfurylamine (FFA) precursor, and their corresponding $\frac{\Delta h/h_{0}}{\Delta T}$ vs temperature plot for (D) AP, (E) MEA, and (F) FFA thin films, respectively. The change from blue to red represents the transition at *T*_v_. Film thickness of all samples is approximately 150 nm.

In summary, this study introduced a simple and efficient ellipsometry-based method to measure the *T*_v_ of vitrimer thin films, offering potential for analyzing complex vitrimer systems. By comparing vitrimer films with permanently crosslinked thermoset systems, we observe a noticeable change in the thermal expansion behaviors of vitrimer films upon surpassing their *T*_v_, as a result of their onset dynamic exchange. We attribute this shift primarily to kinetic factors in vitrimer samples above *T*_v_, including bond-exchange-mediated changes induced internal surface tension and reduced viscosity. Notably, in our work, it is found that catalyst loading ratio and temperature ramping rate did not influence the *T*_v_ measured by ellipsometry, in contrast to conventional thermomechanical-based methods. This distinction could arise from the ability of ellipsometry to directly capture dynamic bond exchange at a microscopic scale without the interference of external forces. Although we understand that apparent *T*_v_ can be dependent on characterization methods and conditions, our method for determining *T*_v_ still offers distinct advantages including eliminating the use of guest molecules and external forces, which the latter can minimize sample deformation. Beyond improving our understanding of the dynamic exchange properties of vitrimers, this method can provide a critical tool for investigating confinement effects on vitrimer film behavior, paving the way for advancements in vitrimer applications in fields like coatings, electronics, and biomedical devices.

## Supplementary Material



## Notes

Detailed experimental procedures, materials and methods; Comprehensive characterization of model vitrimer systems including DGEBA–sebacic acid/TBD (1–5 mol %) and disulfide-based PEGDGE/4-AFD networks; Insoluble content of thin films vs curing time; Ellipsometry data: thickness–temperature plots, refractive index–temperature data, and heating/cooling cycling studies; TMA and nonisothermal creep measurements for bulk samples; Additional analysis of permanently cross-linked network (PDF)
