# Supplementary material for: Ellipsometric Characterization of Network Topology Transition in Vitrimers
Source: ACS Macro Lett. 2025 Nov 14;14(12):1801–7. doi: 10.1021/acsmacrolett.5c00640 (PMC12713773; doi:10.1021/acsmacrolett.5c00640)
Supplement: Supplementary file 1 [file mz5c00640_si_001.pdf]

Supporting Information For:

Ellipsometric Characterization of Network Topology Transition in Vitrimers

Yuming Wang[a], Jaylen Davis[a,b], Travis L. Thornell[c], Sergei Nazarenko[a], Derek L. Patton[a], Yoan C. Simon[d,*], Zhe Qiang[a,*]

[a] School of Polymer Science and Engineering, University of Southern Mississippi, Hattiesburg, 39406, MS, USA
[b] Environmental Laboratory, U. S. Army Engineer Research and Development Center, Vicksburg, 39180, MS USA
[c] Geotechnical and Structures Laboratory, U. S. Army Engineer Research and Development Center, Vicksburg, 39180, MS USA
[d] School of Molecular Sciences, Arizona State University, Physical Sciences Center PSD 104, Tempe, 85287, AZ, USA

Corresponding to: Y.C.S.: Yoan.Simon@asu.edu; Z.Q.: zhe.qiang@usm.edu

Experimental Section

Materials

Bisphenol A diglycidyl ether (DGEBA), sebacic acid, triazabicyclodecene (1,5,7-triazabicyclo [4.4.0] dec-5-ene or TBD), 4,4'-methylenedianiline, 1,4-butanediol diglycidyl ether, Poly(ethylene glycol) diglycidyl ether (PEGDGE 200), 4-Aminophenyl disulfide (4-AFD),ethanol (99%), toluene (99.8%), dichloromethane (DCM, 99.8%), and acetone (99%) were all purchased from Sigma Aldrich. Aminopropyl diethylamine (AP), monoethanolamine (MEA), and furfurylamine (FFA) precursors were prepared according to procedures described in Supporting Information. Silicon wafers were obtained from University Wafer Inc. and used as substrates for depositing thin films. All chemicals were used as received, unless otherwise noted.

Bulk sample preparation

The model vitrimer system was prepared using DGEBA and sebacic acid with the presence of TBD serving as catalyst. For preparing bulk samples, DGEBA (6.8 g, 0.02 mol) was heated in a round-bottom flask at 80 °C under vacuum for 30 min to remove residual water. Subsequently, sebacic acid (4.04 g, 0.02 mol) was introduced into the flask at an increased temperature of 140 °C under stirring. After 15 min, TBD was introduced into the reaction flask in varying amount, specifically 1, 3, and 5 mol% relative to the total molar amount of sebacic acid and DGEBA. The homogenous reaction mixture was then poured into a silicon mold (sample dimension: 50 mm ×4.5 mm × 0.5 mm) and cured at 180 °C for 12 h. The disulfide vitrimer system was prepared using 1:2 molar ratio of 4-afd and PEGDGE 200. For preparing bulk samples, PEGDGE 200 (4 g, 0.02 mol) and 4-AFD (2.48g, 0.01 mol) were mixed in a round-bottom flask with stir bar. After 10 min of stirring, the homogenous mixture was then poured into the same silicon mold. The mixture was cured at 120 °C for 2h and then cured under 150°C for another 2h.

Synthesis of PA-TEG Ester. Phenol-terminated triethylene glycol (PA-TEG-ester) was synthesized via a modified Fischer esterification procedure, based on previously reported methods.[1,2] TEG was dried under reduced pressure to remove any absorbed water prior to use. Generally, TEG (1 eq) was combined with PA (2 eq) and p-TSA (0.5 wt%) in a clean round bottom flask with a stir bar and reacted at 110 °C for 24 h. The crude reaction was allowed to cool slightly and precipitated in diethyl ether to remove any non-hydrolyzed p-TSA to afford a light beige powder. The powder was dried in a vacuum oven at 60 °C overnight to yield the final product. ^{1}H NMR (600 MHz, DMSO-d_6) δ 2.56 (t, exp 3.96H, th 4H), 2.74 (t, exp 3.97H, th 4H), 3.52 (s, exp 4.12H, th 4H), 3.58 (t, exp 3.94H, th 4H), 4.12 (t, exp 4.00H, th 4H), 6.69 (d, exp 4.00H, th 4H), 7.00 (d, exp 4.02H, th 4H), 9.15 (s, exp 2.02H, th 2H). ^{13}C NMR (600 MHz, DMSO-d_6) δ 29.97 (CH_2), 35.98 (CH_2), 63.62 (CH_2), 68.78 (CH_2), 70.21 (CH_2), 115.56 (CH), 129.56 (CH), 130.99 (C), 156. 09 (C), 172.73 (COOH).

Synthesis of MEA-Bz. Monoethanol amine (MEA) functionalized diphenolic ester was prepared by first adding MEA (1eq) dropwise to paraformaldehyde (PFA, 1 eq) in a round bottom flask over ice until the MEA was fully incorporated. The mixture was then heated to 80 °C and allowed to stir for 1 h before the mixture was cooled to 50 °C and vacuum dried to remove water. The freshly prepared triazine (2 eq) was then combined with PA-TEG (3 eq) and PFA (6 eq) in a round bottom flask with chloroform. The reaction was refluxed for 72 hours, following a procedure from literature,[3] to yield the polybenzoxazine monomer. The completed reaction was cooled and filtered to remove any PFA and the organic layer was washed with a 0.1N solution of NaOH to remove any unreacted

phenol and oligomers, followed by water to remove any unreacted amine. The organic layer was dried with $MgSO_4$, filtered, and concentrated to remove the organic solvent. The purified product was dried under vacuum at 50 °C overnight to obtain the final product as a viscous light-yellow liquid. ^1H NMR (600 MHz, $CDCl_3$) δ 2.57 (t, exp 4.29H, th 4H), 2.73 (t, exp 8.09H, th 8H), 3.53 (s, exp 4.22H, th 4H), 3.59 (m, exp 8.30H, th 8H), 3.93 (s, exp 3.75H, th 4H), 4.13 (t, exp 4.00H, th 4H), 4.78 (s, exp 3.80H, th 4H), 6.64 (d, exp 1.73H, th 2H), 6.85 (s, exp 1.75H, th 2H), 6.94 (d, exp 2.01H, th 2H). ^{13}C NMR (600 MHz, $CDCl_3$) δ 30.02 (CH_2) , 35.75 (CH_2), 50.39 (CH_2), 53.89 (CH_2), 60.07 (CH_2), 63.36 (CH_2), 68.79 (CH_2), 70.22 (CH_2), 83.10 (CH_2), 116.16 (CH), 120.78 (C), 127.61 (CH), 127.73 (CH), 132.43 (C), 152.75 (CH), 172.71 (COOH).

Synthesis of AP-Bz. N-(3-aminopropyl)diethanolamine (APDEA) functionalized diphenolic ester was synthesized using a similar procedure as described for MEA-Bz to yield a viscous dark reddish-brown liquid. ^1H NMR (600 MHz, $CDCl_3$) δ 1.68 (t, exp 3.74H, th 4H), 2.61 (m, exp 15.06H, th 16H), 2.80 (m, exp 7.16H, th 8H), 3.60 (m, exp 15.90H, th 16H), 3.90 (s, exp 4.10H, th 4H), 4.19 (t, exp 4.00H, th 4H), 4.77 (s, exp 3.18H, th 4H), 6.64 (d, exp 1.49H, th 2H), 6.75 (s, exp 1.54H, th 2H), 6.89 (d, exp 1.52H, th 2H). ^{13}C NMR (600 MHz, $CDCl_3$) δ 25.07 (CH_2), 30.09 (CH_2), 35.93 (CH_2), 48.81 (CH_2), 50.11 (CH_2), 52.27 (CH_2), 56.18 (CH_2), 59.67 (CH_2), 63.50 (CH_2), 69.14 (CH_2), 70.51 (CH_2), 77.41 (CH_2), 82.02 (CH_2), 116.29 (CH), 119.79 (C), 127.27 (CH), 127.52 (CH), 132.53 (C), 152.37 (C), 172.88 (COOH).

Synthesis of FFA-Bz. Furfurylamine (FFA) functionalized benzoxazine was synthesized using a similar procedure as mentioned for MEA-Bz to yield a viscous red liquid. ^1H NMR (600 MHz, $CDCl_3$) δ 2.58 (t, exp 4.07H, th 4H), 2.82 (t, exp 4.11H, th 4H), 3.60 (m, exp 8.16H, th 8H), 3.87 (s, exp 3.57H, th 4H), 3.95 (s, exp 3.40H, th 4H), 4.22 (m, exp 4.16H, th 4H), 4.80 (s, exp 3.56H, th 4H), 6.21 (s, exp 2.11H, th 2H), 6.30 (s, exp 2.18H, th 2H), 6.71 (d, exp 1.97H, th 2H), 6.75 (s, exp 1.73H, th 2H), 6.92 (d, exp 1.94H, th 2H), 7.37 (s, exp 2.00H, th 2H). ^{13}C NMR (600 MHz, $CDCl_3$) δ 30.11 (CH_2) , 35.92 (CH_2), 48.16 (CH_2), 49.56 (CH_2), 63.48 (CH_2), 69.15 (CH_2), 70.51 (CH_2), 81.72 (CH_2), 108.89 (CH), 110.23 (CH), 116.46 (CH), 119.53 (C), 127.31 (CH), 127.63 (CH), 132.71 (C), 142.52 (CH), 151.69 (C), 151.36 (C), 172.78 (COOH).

Thin film sample preparation

To prepare vitrimer thin films, silicon wafers (substrate thickness: 600-725 μm) were first cut into square shape with a size of approximately 1.5 cm × 1.5 cm and treated with a UV-ozone cleaner (SC-UV-I UV Ozone Cleaner) for 2 h, allowing the removal of organic impurities and oxidation of the native silicon layer. Polymer solutions were prepared by dissolving precursors in different solvents at a concentration of 5 wt%. Specifically, acetone was used to dissolve sebacic acid-DGEBA system (1:1 of acid: epoxy in molar ratio) and disulfide system (1:2 of amine: epoxy in molar ratio), and DCM was used to dissolve precursors of polybenzoxazine-based vitrimers (including AP, MEA, and FFA). Precursors for the permanently crosslinked networks, including 1,4-butanediol diglycidyl ether and 4,4'-methylenedianiline, were dissolved in a DCM-toluene co-solvent mixture at a 9:1 mass ratio. In this work, a MicroNano Tools KW-4A spin coater was used for preparing polymer thin films. Specifically, a polymer solution was applied to the substrate and spun at 200 rpm for 10 s, followed by an increase to 4000 rpm for another 10 s to form thin films. Subsequently, thin films including AP, MEA, FFA, and the model DGEBA-sebacic acid vitrimer system were cured at 180 °C for 15 min - 1 h. Disulfide system were cured under 150 °C for 30 min

– 4 h.

Sample characterization

Fourier transform infrared spectroscopy (FTIR) was used to characterize the chemical composition of both bulk and film samples, using Nicolet 6700 (Thermo Fisher) and averaging over 32 scans with a resolution of 1 cm^{-1}. Thermal stability of vitrimers was determined by thermogravimetric analysis (TGA) using a thermogravimetric analyzer Q50 (TA Instruments); approximately 10 to 15 mg of samples were heated under nitrogen atmosphere at 10 °C/min until 600 °C, and the temperature at which 5% mass loss occurred was determined as the polymer thermal decomposition temperature (T_{d5}). The glass transition temperature (T_g) of bulk vitrimer samples were characterized by differential scanning calorimetry (DSC) using TA Instruments Discovery DSC250. A heat−cool−heat cycle was applied with a temperature range from 0 to 200 °C and a ramping rate of 10°C/min to remove thermal history; T_{g} was determined by the midpoint of an endothermic stepwise change in the plot. Additionally, the thermal curing for vitrimer precursors was also characterized by DSC measurements, with temperature ranging from 0 °C to 240 °C and a ramp rate of a 5 °C/min. TMA were performed using Discovery TMA 450 RH with expansion probe. Materials were heated from room temperature with 5 °C/min heating speed and 0.02N force loaded. Non-isothermal creep measurements were performed as a conventional approach to determine T_{v} of bulk vitrimer samples, using Discovery dynamic mechanical analyzer (DMA) 850 from TA Instruments. In these measurements, sample bars prepared from using a silicon mold (sample dimension: 30.0 mm × 5.00 mm × 1.00 mm) were first subjected to a pre-load force of 0.01 N. After equilibrium was reached, the stress was increased to different levels ranging from 2 kPa to 50 kPa, and the temperature was changed from room temperature to 300 °C at a heating rate of 5 °C/min.

For thin film characterization, spectroscopic ellipsometry (J.A.Woollam M-2000) was employed to determine the change of film thickness as a function of temperature under high purity nitrogen gas (> 99.9%), which allowed us to identify their thermal transitions. Ellipsometry measurements on vitrimer thin films were performed at an incidence angle of 70° with a wavelength range from 500 to 1600 nm. The amplitude change (Ψ) and phase change (Δ) were measured every 1 s and fitted using a Cauchy model to obtain film thickness (h) and refractive index (n). The polymer film was placed on a sample stage, undergoing a heat and cool ramp with a ramp rate of 5 °C/min. To quantitatively determine T_{g}, we followed an established method by converting the temperature-thickness relationship through the cooling ramp.[49] Using equation (1) below, thickness-temperature coefficient can be determined and plotted against temperature.

$$\frac{\Delta h/h_0}{\Delta T} = \frac{h\left(T+\frac{\Delta T}{2}\right)-h\left(T-\frac{\Delta T}{2}\right)}{h_0\Delta T}, \qquad (1)$$

where $\frac{\Delta h/h_0}{\Delta T}$ is the temperature dependent normalized apparent thermal expansivity of polymer thin films, h is the temperature-dependent thickness, h_0 is the initial polymer film thickness, and ΔT is the differentiation range, which was set to be 5 °C.[50] In some cases, this temperature dependent thermal expansivity could be interpreted as apparent thermal expansion coefficient of polymer samples and noted as α(T) . However, in this study, we refer to it as $\frac{\Delta h/h_0}{\Delta T}$, since the measured values from vitrimer thin films are likely convolved with contributions from both thermal expansion and relaxation-mediated changes in surface tension. In the $\frac{\Delta h/h_0}{\Delta T}$ vs. T plot, there is a step change in

$\frac{\Delta h/h_0}{\Delta T}$ that corresponds to the glass transition, occurring between the nearly temperature-independent rubbery plateau and the glassy state with a much smaller $\frac{\Delta h/h_0}{\Delta T}$. The midpoint of this transition is considered the glass transition temperature (T_g) as obtained from ellipsometry. For maximum curing condition tests, maximum ramp temperature is 100°C to avoid potential reaction. For determining network topology transition and associated T_v, linear extrapolations were drawn across distinct regions (solid-like to flow-like) at elevated temperatures, and their intersection was taken as T_v. These values are then labeled on the corresponding plots of $\frac{\Delta h/h_0}{\Delta T}$ as a function of temperature. Moreover, insoluble content of films after crosslinking were determining by immersing them in acetone for 5 min, after which the films were removed from the solvent and dried completely under vacuum at 70 °C overnight. The thickness of polymer films was measured by ellipsometry before and after solvent extraction and then insoluble content (%) can be calculated using the following equation (2), where h_b represent the film thickness before solvent extraction and h_a represent film thickness after extraction.

$$\text{Insolube content} = \frac{h_a}{h_b} * 100\% \ , \qquad (2)$$

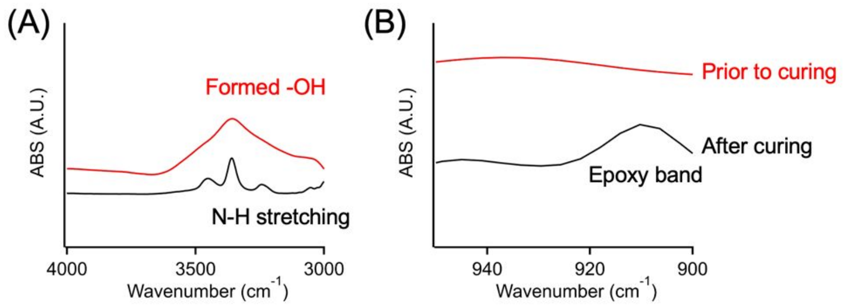

Figure S1. FTIR results of disulfide-based vitrimer, prepared by crosslinking of PEGDGE with 4-AFD, prior to and after curing at 150 °C for 1 h.

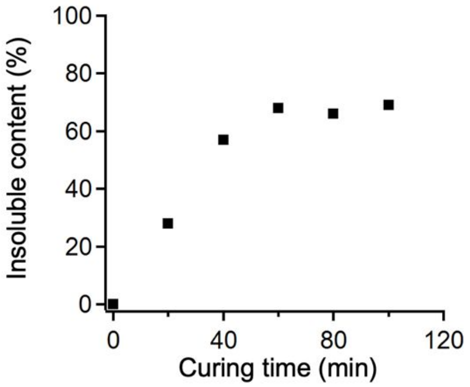

Figure S2. Insoluble content of disulfide vitrimer film as a function of curing time at 150 °C.

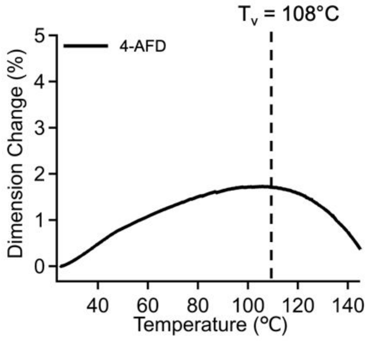

Figure S3. TMA result showing the change in sample dimension of disulfide vitrimer as a function of temperature. The applied stress was 0.02 N and temperature ramp was 5 °C/min.

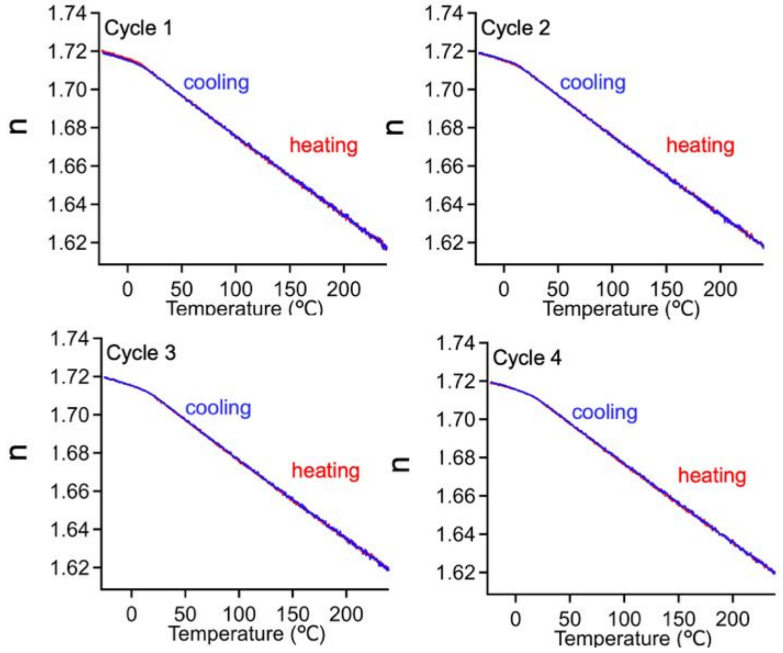

Figure S4. Refractive index (n) as a function of temperature for disulfide-based vitrimer films obtained during cooling (blue) and heating (red) cycles over multiple thermal cycles.

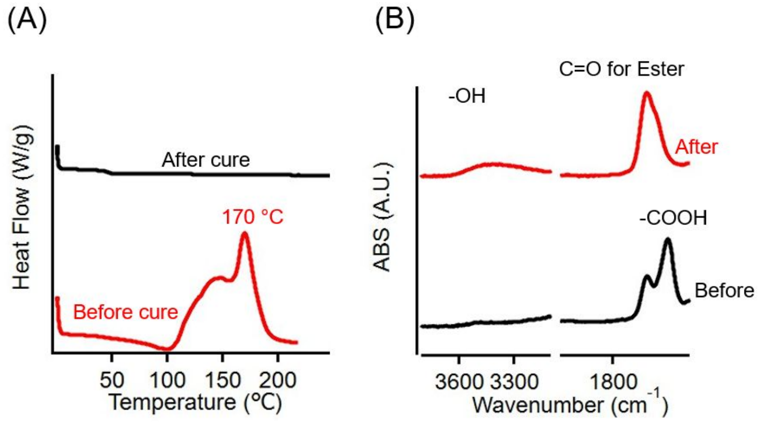

Figure S5. (A) DSC results for vitrimer precursor (DEGBA-sebacic acid mixture with 5 mol% TBD) before and after cure under 180°C for 12h and (B) their corresponding FTIR results prior to and after curing.

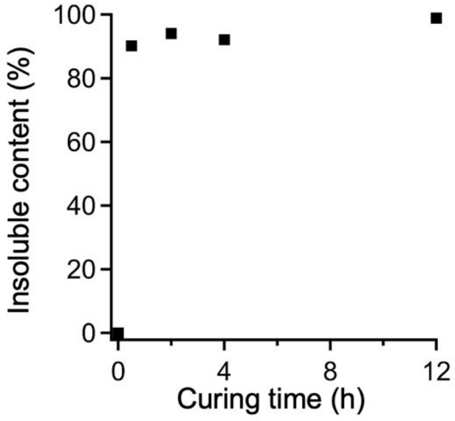

Figure S6. Insoluble content of DGEBA-sebacic acid vitrimer film as a function of curing time with the presence of 5 mol% TBD.

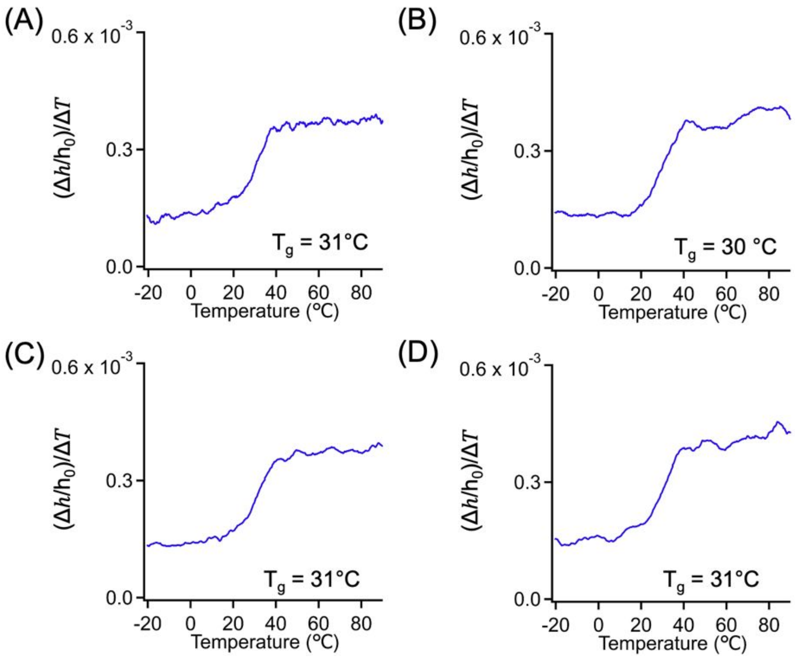

Figure S7: $\frac{\Delta h/h_0}{\Delta T}$ versus temperature for DGEBA–sebacic acid vitrimer films containing 5 mol % TBD after curing at 180 °C for (A) 15 min, (B) 30 min, (C) 1 h, and (D) 2 h. The midpoint of each step-like transition is taken as the Tg determined by ellipsometry.

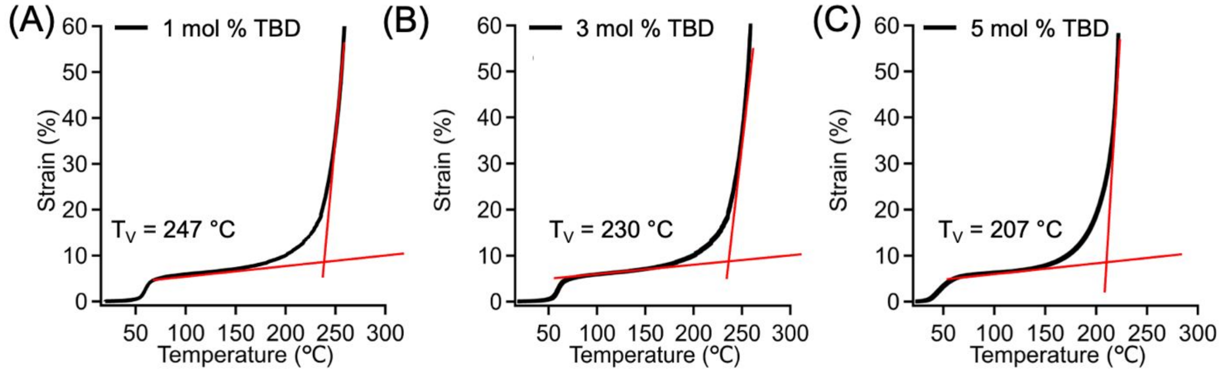

Figure S8. Non-isothermal creep measurements for DGEBA-sebacic acid vitrimer with (A) 1 mol%, (B) 3 mol%, (C) 5 mol% TBD. The applied stress in these measurements was 15 kPa.

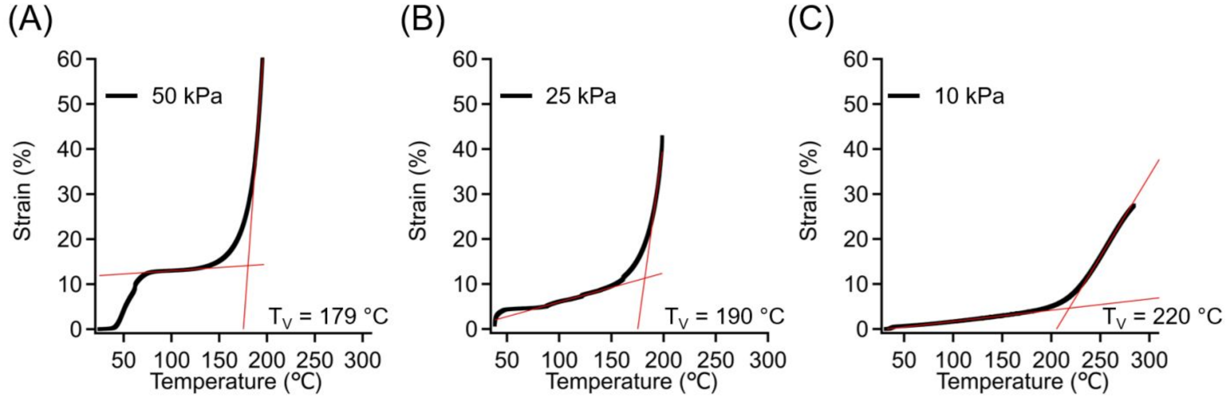

Figure S9. Non-isothermal creep results for 5mol% TBD loaded DGEBA-sebacic acid vitrimer system with a constant stress of (A) 50 kPa (B) 25 kPa and (C) 10 kPa.

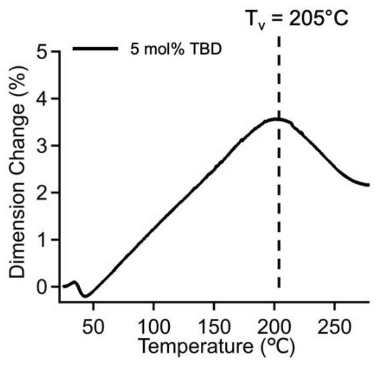

Figure S10. TMA result showing the change in sample dimension of 5mol% TBD loaded DGEBA-sebacic acid vitrimer system as a function of temperature. The applied stress was 0.02 N and temperature ramp was 5 °C/min.

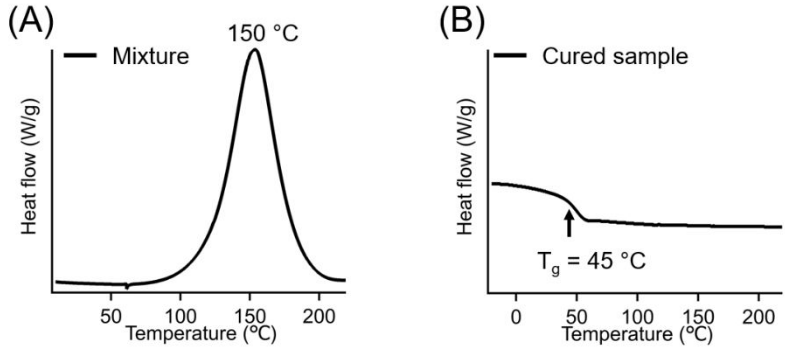

Figure S11. (A) DSC for mixture of 4,4'-methylenedianiline and 1,4-butanediol diglycidyl ether before curing and (B) after curing at 170 °C for 2h.

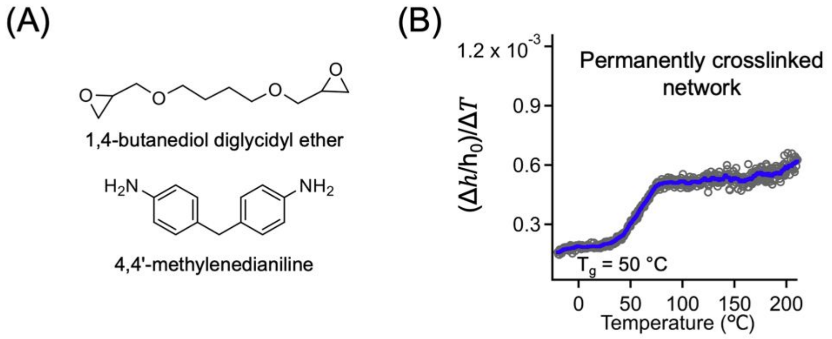

Figure S12. (A) Chemicals for preparing a permanently crosslinked network, and (B) their derived $\frac{\Delta h/h_0}{\Delta T}$ versus temperature plot. Film thickness is approximately 150 nm.

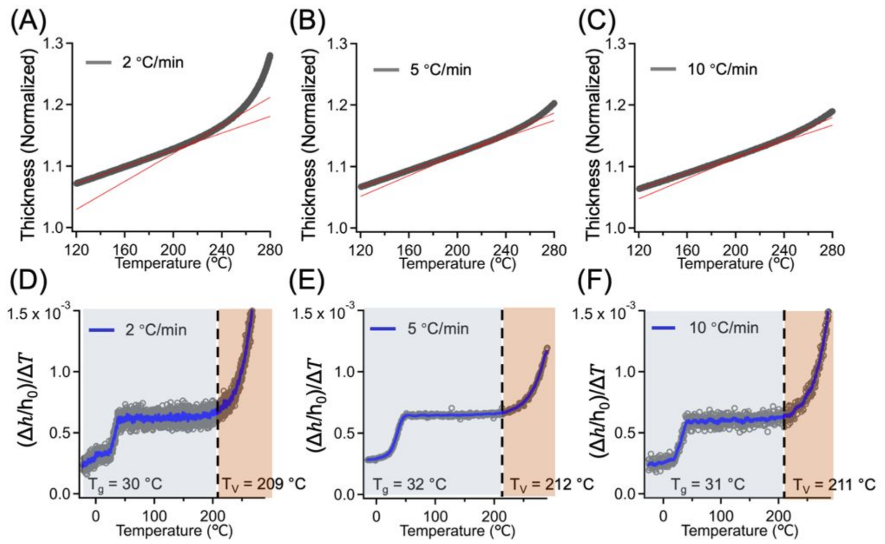

Figure S13. Thickness-temperature plot for 5mol%TBD loaded DGEBA-sebacic acid films with a temperature ramp of (A) 2 °C/min (B) 5 °C/min (C) 10 °C/min and $\frac{\Delta h/h_0}{\Delta T}$ versus temperature plot for same systems with temperature ramp of (D) 2 °C/min (E) 5 °C/min (F) 10 °C/min.

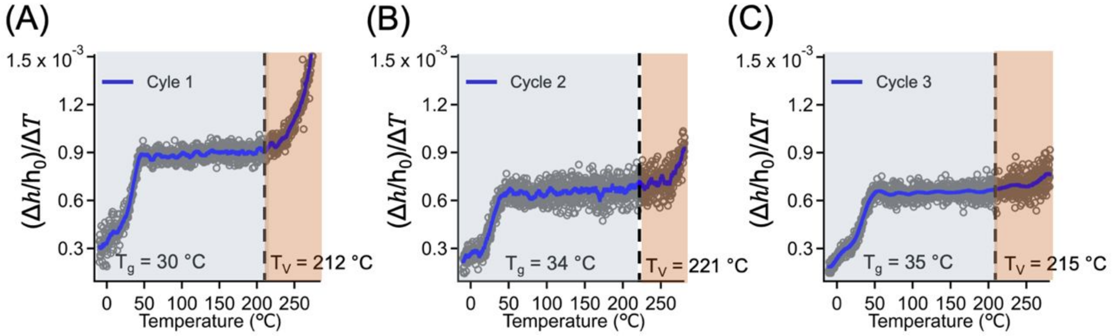

Figure S14. $\frac{\Delta h/h_0}{\Delta T}$ versus temperature plot for DGEBA-sebacic acid films with 5 mol% TBD after multiple cycles, including (A) cycle 1 (B) cycle 2 and (C) cycle 3.

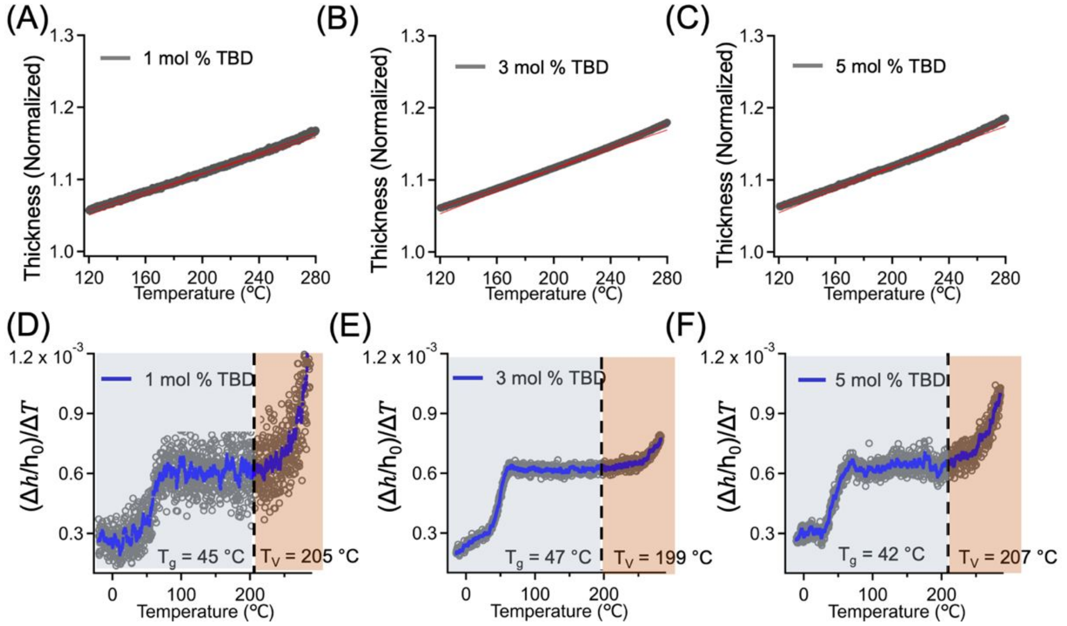

Figure S15. Thickness-temperature plot for DGEBA-sebacic acid films (prepared from dip-casting) with (A) 1 mol% (B) 3 mol% (C) 5 mol% TBD and $\frac{\Delta h/h_0}{\Delta T}$ versus temperature plot for the same systems with (D) 1 mol% (E) 3 mol% (F) 5 mol% TBD.

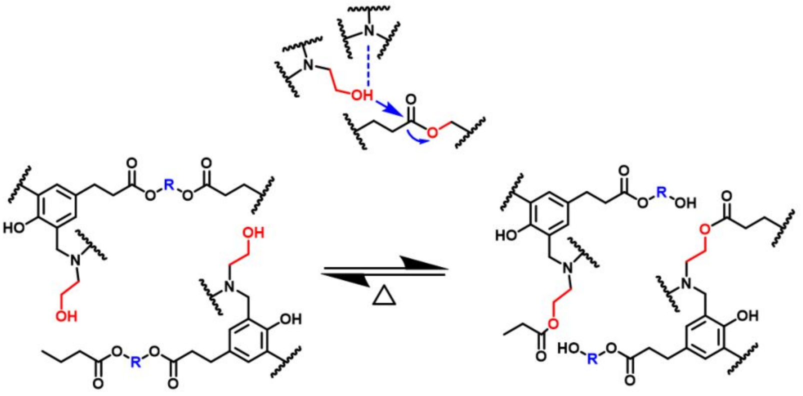

Figure S16. Dynamic exchange mechanism of benzoxazine-based vitrimers in this study, using MEA as an example.

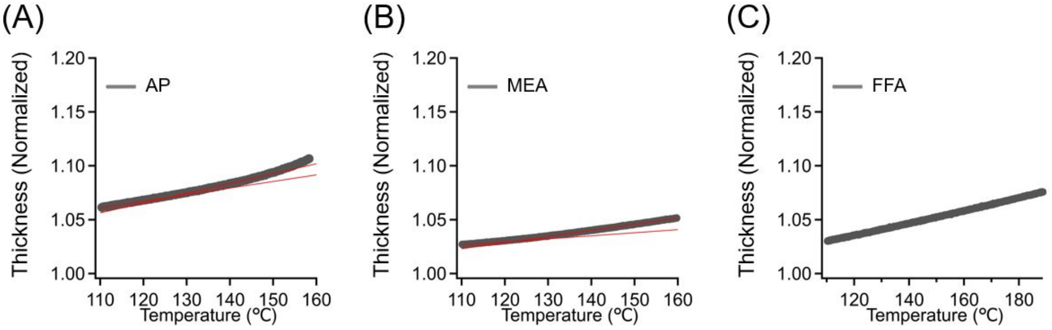

Figure S17. Thickness-temperature plot for (A)AP, (B)MEA and (C)FFA systems

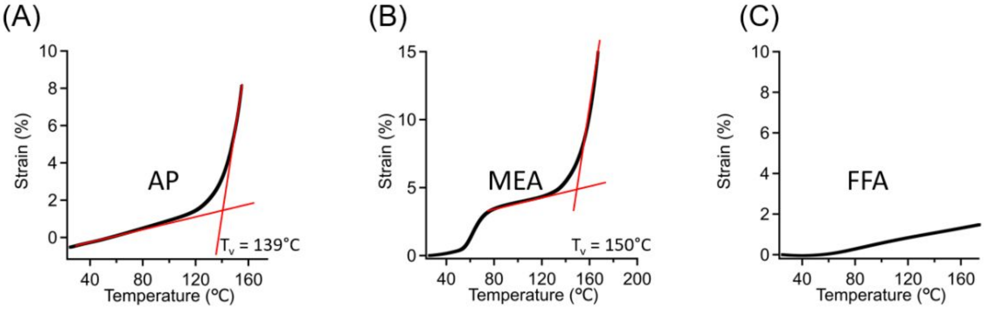

Figure S18. Non-isothermal creep results for (A)AP, (B)MEA and (C)FFA

References

(1) Adjaoud, A.; Trejo-Machin, A.; Puchot, L.; Verge, P. Polybenzoxazines: a sustainable platform for the design of fast responsive and catalyst-free vitrimers based on trans-esterification exchanges. *Polymer Chemistry* **2021**, *12* (22), 3276-3289.

(2) Trejo-Machin, A.; Verge, P.; Puchot, L.; Quintana, R. Phloretic acid as an alternative to the phenolation of aliphatic hydroxyls for the elaboration of polybenzoxazine. *Green Chemistry* **2017**, *19* (21), 5065-5073.

(3) Men, W.; Lu, Z.; Zhan, Z. Synthesis of a novel benzoxazine precursor containing phenol hydroxyl groups and its polymer. *Journal of applied polymer science* **2008**, *109* (4), 2219-2223.
